# Efectividad de una Estrategia de enseñanza en Administración de Medicamentos en Pediatría

**DOI:** 10.15649/cuidarte.2042

**Published:** 2021-08-20

**Authors:** Mery Luz Valderrama Sanabria

**Affiliations:** 1 Universidad de los Llanos, Villavicencio, Colombia. Email: mvalderrama@unillanos.edu.co Universidad de los Llanos Universidad de los Llanos Villavicencio Colombia mvalderrama@unillanos.edu.co

**Keywords:** Educación en Enfermería, Rendimiento académico, Evaluación Educacional., Education, Nursing, Academic Performance, Educational Measurement, Educação em Enfermagem, Desempenho Acadêmico, Avaliação Educacional.

## Abstract

**Introducción:**

La pandemia originada por el nuevo coronavirus, hizo que los docentes universitarios enfrentaran el desafío de adoptar nuevas estrategias didácticas de tipo virtual para dar continuidad al proceso enseñanza-aprendizaje. El objetivo del estudio fue determinar la efectividad del uso de un objeto virtual de aprendizaje en la formación de profesionales de Enfermería.

**Materiales y Método:**

Se trata de un estudio de tipo cuantitativo cuasi experimental pre-post con grupo control sin aleatorización. Las mediciones estuvieron orientadas a determinar el efecto de la aplicación del objeto virtual de aprendizaje

**Resultados:**

La población estuvo constituida por la totalidad de estudiantes de quinto semestre de Enfermería de la Universidad de los Llanos, durante el primer y segundo semestre de 2018. Se garantizó una muestra de 81 sujetos (40 del grupo control y 41 del experimental). Se utilizó prueba de McNemar para determinar diferencias entre grupos pareados y la U de Mann Whitney para comparar los puntajes y la diferencia. Se evidenció el desarrollo de competencias en los dos grupos, pero con mayor nivel en el grupo intervenido con el objeto virtual, lo cual indica que la estrategia mejoró significativamente el desarrollo de competencias en comparación con la enseñanza tradicional y fue de bastante utilidad en época de pandemia.

**Conclusiones:**

La aplicación del objeto virtual de aprendizaje favoreció el proceso enseñanza aprendizaje, permitió desarrollar la competencia de administrar medicamentos en pediatría, de forma crítica y reflexiva.

## Abstract (en)

**Introduction:**

The pandemic originated by the new coronavirus, made university teachers face the challenge of adopting new didactic online strategies to give continuity to the teaching- learning process.

**The objective:**

of the study was to determine the effectiveness in the use of an online learning object in the nursing professionals training.

**Materials and Methods:**

This is a quantitative quasi-experimental pre-post study with a non-randomized control group. The measurements were aimed at determining the effect of the online learning object application.

**Results:**

The population was made up by all fifth-semester Nursing students of the Universidad de los Llanos, during the first and second semester of 2018. A sample of 81 subjects (40 in the control group and 41 in the experimental group) was guaranteed. The McNemar test was used to determine differences between paired groups and Mann Whitney U to compare scores and difference. The skills development was evidenced in both groups, but with a higher level in the group intervened with the online object, which indicates that the strategy significantly improved the development of skills compared to traditional teaching and was quite useful in times of pandemic.

**Conclusions:**

The application of the online learning object favored the teaching-learning process, allowed developing the competence of administering medications in pediatrics, in a critical and reflective manner.

## Resumo

**Introdução:**

A pandemia originada pelo novo coronavírus fez com que professores universitários enfrentassem o desafio de adotar novas estratégias didáticas de tipo virtual para dar continuidade ao processo de ensino-aprendizagem.

**O objetivo:**

do estudo era determinar a eficácia do uso de um objeto de aprendizagem virtual no treinamento de profissionais de Enfermagem. Materiais e métodos: Este é um estudo quantitativo quasi-experimental pré e pós com um grupo de controle não aleatório. As medidas tinham como objetivo determinar o efeito da aplicação do objeto virtual de aprendizagem.

**Resultados:**

A população consistia de todos os estudantes de Enfermagem do quinto semestre da Universidad de los Llanos, durante o primeiro e segundo semestres de 2018. Uma amostra de 81 sujeitos foi garantida (40 do grupo de controle e 41 do grupo experimental). O teste McNemar foi usado para determinar diferenças entre grupos emparelhados e o teste U de Mann-Whitney para comparar pontuações e diferenças. Houve desenvolvimento de competências em ambos os grupos, mas com um nível mais alto no grupo que participou com o objeto virtual, indicando que a estratégia melhorou significativamente o desenvolvimento de competências em comparação ao ensino tradicional e foi bastante útil em tempos de pandemia.

**Conclusões:**

A aplicação do virtual objeto de aprendizagem favoreceu o processo de ensino-aprendizagem, permitiu o desenvolvimento da competência de administração de medicamentos em pediatria, de forma crítica e reflexiva.

## Introducción

Con ocasión de la pandemia originada por el nuevo coronavirus, los docentes universitarios se enfrentaron al desafío de adoptar nuevas estrategias didácticas de tipo virtual para las que no estaban preparados(1)-(2), en este caso las principales acciones están orientadas a la investigación asistida por las TIC sin olvidar la flexibilidad en el proceso de enseñanza, al tener en cuenta el ritmo particular de aprendizaje de cada estudiante(1). Es así como el profesor acopló los contenidos y programa curricular mostrando un desarrollo satisfactorio en las competencias digitales que debe poseer, al utilizar diversas plataformas con el fin de cumplir los objetivos académicos(3).

Como hecho positivo, se observó una actitud proactiva hacia las tecnologías de la información y comunicación, mostrando un enfoque innovador como producto de la emergencia sanitaria decretada en donde el docente universitario desarrolló sus competencias digitales de manera satisfactoria al entorno de la crisis mundial en un nuevo escenario educativo(3).

Una de las estrategias empleadas fue el El Objeto Virtual de Aprendizaje (OVA) que hace referencia a un mecanismo de aprendizaje autorregulado, en el que estudiante tiene la capacidad de controlar su propio aprendizaje, participando de manera reflexiva(4). Para enfermería y la administración de medicamentos en pediatría resulta ser una estrategia innovadora, que ofrece variedad de ayudas audiovisuales como videos, fotos, audios y presentaciones con las cuales el futuro profesional va reconociendo sus capacidades, utiliza aprendizajes previos, se compromete a entregar tareas y se encuentra constantemente motivado.

Ahora bien, entrando en detalle, es trascendental reconocer que el futuro profesional de Enfermería se enfrentará a una sociedad en constante cambio. Por tanto, debe estar preparado para asumir riesgos y responder a las necesidades del mercado laboral. En esta misión, el profesor asume un rol indispensable para contribuir en la formación de talento humano que sea capaz de enfrentar retos. El docente influye de manera directa en la calidad del proceso enseñanza-aprendizaje y debe replantear su quehacer(5). Así pues, la calidad de la formación universitaria se convierte en una prioridad de las instituciones de educación(6)-(8). Se requiere el uso de estrategias didácticas que favorezcan el desarrollo de competencias y propicien la toma de decisiones asertivas y útiles a lo largo de la vida personal y laboral(9).

La administración de medicamentos es una responsabilidad legal del profesional de enfermería. Por tanto, son indispensables los conocimientos para cumplir esta labor de manera eficiente y responsable a través de la promoción de nuevas estrategias para los profesionales que se están formando(10). Una de las competencias más importantes en el currículo de enfermería es la administración segura de medicamentos. No obstante, siguen siendo comunes los errores en el resultado de prácticas débiles que afectan la calidad de este procedimiento ejercido por el estudiante, como también es cierto que en el caso específico de pediatría se considera un factor estresor adicional para el alumno(11).

Algunos investigadores sugieren diseñar estrategias formativas que mejoren el intelecto relacionado con la dosificación de los medicamentos en pediatría(12),(13). Gran proporción de errores en la medicación, ocurren por fallo en los cálculos matemáticos, particularmente en la población pediátrica, siendo el riesgo de error en el momento de la preparación del 26 al 60%, debido a que hay enfermeras que no comprenden con claridad las operaciones matemáticas básicas como suma, resta, multiplicación y división. Por tanto, no logran aplicarlas al concepto de cálculo de dosis y aplicación de fórmulas, así como también se presentan dificultades con las fracciones, porcentajes y equivalencias(14)-(15).

El cálculo de la dosis de un medicamento puede ser de simple a complejo dependiendo el número de conversiones matemáticas que se requieran. El estudiante de enfermería debería adquirir la competencia desde la práctica formativa. Por eso es fundamental que fortalezca las habilidades matemáticas y conozca la manera correcta de establecer una dosis incluyendo sus fracciones(16). Por lo anteriormente expuesto, se planteó como objetivo, determinar la efectividad del uso de un objeto virtual de aprendizaje en la formación de profesionales de Enfermería de la Universidad de los Llanos, Colombia.

## Materiales y Métodos

Inicialmente, se diseñó el Objeto Virtual de Aprendizaje que es un material estructurado de forma significativa, asociado a un propósito educativo con carácter digital(17). Se contó con una etapa pedagógica en la cual se fijó el objetivo, contenidos y actividades de aprendizaje. Y en la etapa tecnológica se definieron los requerimientos funcionales, diseño gráfico y computacional. El OVA estuvo constituido por cinco módulos que incluían ejercicios de pensamiento lógico matemático, correctos en la administración de medicamentos, cálculo de dosis pediátrica, cálculo de velocidad de volumen de infusión, farmacodinamia, cálculo de necesidades de líquidos y detección de errores. Se implementó en la plataforma Moodle de la Universidad de los Llanos.

Se trata de un estudio cuantitativo cuasi experimental pre-post con grupo control sin aleatorización. El grupo de control estuvo conformado por los estudiantes matriculados en el primer período académico de 2018 en el curso de “Cuidado de la Salud al Niño” que pertenecían a quinto semestre del programa de Enfermería de la Universidad de los Llanos. El grupo experimental estuvo conformado por los estudiantes matriculados en el segundo período académico de 2018 en quinto semestre.

### Población y muestra

La población estuvo constituida por la totalidad de estudiantes que se encontraban cursando quinto semestre en el Programa de Enfermería de la Universidad de los Llanos, durante el primer y segundo semestre de 2018. En cuanto al tamaño muestral, fue factible acceder al total de alumnos matriculados en cada uno de los semestres, 81 sujetos (40 del grupo control y 41 del experimental).

Como criterios de inclusión se estableció al estudiante hombre o mujer mayor de 18 años perteneciente a quinto semestre, matriculado por primera vez en el curso de “Cuidado de la Salud al Niño” en los períodos establecidos.

### Recolección de los datos

Se realizó de marzo de 2018 a marzo de 2019 mediante la aplicación del Examen Clínico Objetivo Estructurado ECOE. Este instrumento fue diseñado y validado por Harden, Stevenson y Downie(18). Es un elemento altamente conocido y utilizado para la evaluación de competencias clínicas del estudiante, tanto de medicina como de enfermería. Consiste en una serie de estaciones de evaluación que, con diferentes metodologías, evalúa la habilidad clínica de un estudiante o profesional en determinada área. Para los dos grupos (control e intervención), se ajustó un ECOE que midiera la competencia administración de medicamentos en pediatría, el cual contó con un Comité de Prueba ad-hoc responsable constituido por expertos en la materia y encargado de definir los componentes de la competencia a evaluar y los criterios de ponderación.

**Figura 1:**
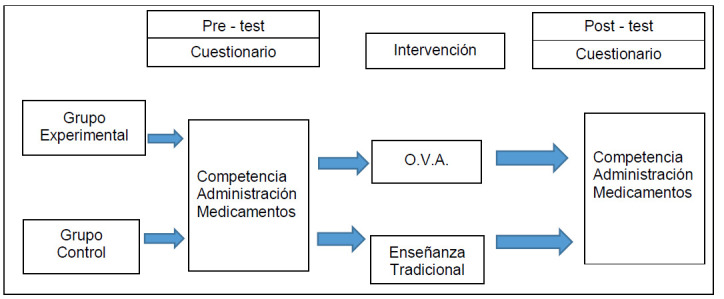
Desarrollo del estudio experimental

Para el análisis se seleccionó el paquete estadístico SPSS versión 24. Se determinó el nivel de significancia, se utilizó la prueba de McNemar(19) para analizar datos binarios apareados y probar la efectividad de los tratamientos con diseños de antes y después, en los mismos individuos, la población no tuvo distribución normal porque se encontraron variables inferiores a 0,05 que se consideraron como no normales. Por tanto, se empleó la prueba por resultados de U de Mann Whitney(20) con el fin de comparar los puntajes y la diferencia.

La investigación fue aprobada por el comité de bioética de la Universidad de los Llanos y hace parte de una tesis doctoral. Todos los participantes firmaron el consentimiento informado.

**Tabla 1:** Variables de estudio

Competencia	Dimensión de la Competencia
Cognitiva	Pensamiento lógico matemático, Cálculo de dosis correcta de medicamento, Mecanismo de acción, Cálculo de volumen de infusión, Cálculo de velocidad de infusión y Efectos adversos de la medicación.
Praxiológica	Información sobre la condición de salud del niño, Administración correcta del medicamento, Administración de la dosis correcta, Paciente correcto, Hora correcta, Administración por la vía correcta, Velocidad correcta, Alergias e interacciones farmacológicas, Aplicación de técnica aséptica y normas de bioseguridad, Rotulación del medicamento a administrar, Desechos de residuos generados en el procedimiento y Registro correcto del medicamento administrado.
Actitudinal	Seguridad durante el proceso de administración de medicamentos, Empatía y compromiso en el acto de cuidar, Autonomía y actitud crítica y asertiva y Presentación personal.
Comunicativa	Trato cordial con el niño, padres y/o cuidador, Orientación y educación sobre el tratamiento farmacológico, Liderazgo y trabajo en equipo.

Fuente: Elaboración propia

## Resultados

Para efectos de este estudio se garantizó una muestra de 81 sujetos (40 del grupo control y 41 del experimental), del grupo control el 87,5% correspondió al género femenino y el 12.5% al masculino, con una media de edad de 20 años y un rango entre 18 y 25 años. Por su parte, el grupo experimental estuvo conformado así: 70.7% del género femenino y 29.3% del masculino, con media de 20 años, la edad osciló entre 18 y 25 años.

Al grupo control, se le realizó una prueba previa de conocimientos. Luego recibieron la clase tradicional, la cual consistió en dos horas de teoría impartida por el docente titular del curso, posteriormente se solicitaba dividirse en grupos conformados por cuatro estudiantes. Se obtuvo el apoyo de docentes para estar pendiente de dos grupos. Se entregó un taller de medicamentos en el que reposaban situaciones clínicas en las que debían realizar el cálculo de dosis, velocidad de infusión, pensamiento lógico matemático y volumen de infusión. En caso de dudas frente al ejercicio, los estudiantes le preguntaban a un compañero o a la profesora, disponían de dos horas para resolver los casos. Al día siguiente en el horario habitual del curso, se dirigían al laboratorio de simulación para seguir practicando las tareas en cuatro horas más. Se asignó otra fecha para la evaluación final por medio del examen clínico objetivo estructurado.

Con el grupo experimental, se desarrollaron los mismos contenidos en el mismo tiempo, tan solo que se intervino la variable enseñanza-aprendizaje por medio del Objeto Virtual de Aprendizaje OVA. Se determinó que la población de estudiantes en los dos grupos sustentó variables demográficas muy similares, por lo cual se consideró viable la inclusión del 100 % de los sujetos. Se excluyeron dos estudiantes del grupo control por estar repitiendo el curso y dos del grupo experimental, uno por ser repetidor y el otro porque canceló la materia.

**Tabla 2:** Resultados de la evaluación en el grupo de participantes.

Variable	Intervenido n:41		Valor p	Control n:40		Valor p
	Pre	Pos		Pre	Pos	
Calculo Dosis	48.8(20)	78.0(32)	0.169	75.0(30)	90.0(36)	<0.001
Informacion	12.2(5)	78.0(32)	0.716	15.0(6)	32.5(13)	0,003
Dosis Correcta	61.0(25)	82.9(34)	0.015	67.5(27)	87.5(35)	0.02
Paciente Correcto	73.2(30)	95.1(39)	<0.001	72.5(29)	90.0(36)	<0.001
Vía Correcta	82.9(34)	97.6(40)	<0.001	52.5(21)	97.5(39)	0,012
Velocidad Correcta	14.6(6)	73.2(30)	0.620	15.0(6)	67.5(27)	0.443
Alergias	34.1(14)	43.9(18)	0.233	37.5(15)	25.0(10)	0.017
Técnica Aséptica	24.4(10)	58.5(24)	0.419	15.0(6)	25.0(10)	<0.001
Rotulación	2.4(1)	68.3(28)	0.182	15.0(6)	25.0(10)	<0.001
Desecho	46.3(19)	82.9(34)	0.141	20(8)	82.5(33)	1.000
Seguridad	36.6(15)	58.5(24)	0.888	92.5(37)	70(28)	<0.001
Presentación Personal	73.2(30)	97.6(40)	<0.001	85.0(34)	95.0(38)	<0.001
Saludo	70.7(29)	95.1(39)	<0.001	95.0(38)	85.0(34)	<0.001
Educación	19.5(8)	68.3(28)	0.609	32.5(13)	57.5(23)	0.672

Fuente: Elaboración propia, prueba de McNemar

Se utilizó la prueba de McNemar para determinar diferencias entre grupos pareados. Particularmente las variables dosis correcta, paciente correcto, vía correcta, presentación personal y saludo aumentaron significativamente del pre al post en el grupo de intervención.

En lo que respecta a la pre-prueba, los estudiantes lograron responder en forma correcta de manera similar en los dos grupos, con cierta ventaja en el grupo control. Sin embargo, el nivel de las competencias estaba por debajo del deseado.

**Tabla 3:** Diferencias significativas entre los grupos.

Variable	Total	Intervenido	Control	Valor p
	(81)	(41)	(40)	
Género Femenino	79.0(64)	70.7(29)	87.5(35)	0.06
Pensamiento Lógico Pos (Cognitiva)	70.4(57)	87.8(36)	52.5(21)	<0.01
Mecanismo de Acción Pos (Cognitiva)	42.0(34)	51.2(21)	32.5(13)	0.09
Dilución, Volumen y Velocidad Infusión Pos (Cognitiva)	59.3(48)	63.4(26)	55.0(22)	0.44
Velocidad Infusión Pos (Praxiológica)	37.0(30)	36.6(15)	37.5(15)	0.93
Volumen Infusión Pos (Praxiológica)	44.4(36)	41.5(17)	47.5(19)	0.58
Efecto Adverso Pos (Praxiológica	34.6(28)	43.9(18)	25.0(10)	0.07
Medicamento Correcto Pos (Praxiológica)	82.7(67)	82.9(34)	82.5(33)	0.96
Hora Correcta Pos (Praxiológica)	96.3(78)	100(41)	92.5(37)	0.12
Registro Pos (Praxiológica)	58.0(47)	82.9(34)	32.5(13)	<0.01
Empatía Pos (Comunicativa y actitudinal)	93.8(76)	97.6(40)	90.0(36)	0.2
Autonomía Pos (Comunicativa y actitudinal)	81.5(66)	78.0(32)	85.0(34)	0.42
Liderazgo y Trabajo en equipo Pos (Comunicativa y actitudinal)	79.0(64)	70.7(29)	87.5(35)	0.06

Fuente: Elaboración propia. Prueba de McNemar

La competencia con menor puntuación en el grupo control fue la praxiológica con las dimensio- nes: mecanismo de acción 32.5% de aciertos, velocidad de infusión 37.5%, efecto adverso 25% y registro 32.5%. Con relación al grupo experimental, la dimensión pensamiento lógico matemá- tico que hace parte de la competencia cognitiva, se obtuvo el 87.8% de respuestas correctas. Se ha discutido la utilidad de la educación matemática con relación al énfasis de la comprensión conceptual, algunos educadores en matemáticas creen que es necesario hacer hincapié en la memoria del procedimiento matemático, mientras que otros creen que los estudiantes primero deben comprender la lógica matemática antes de aplicar el concepto(21). En la competencia pra- xiólogica se destacaron la hora correcta con 100% y el registro con 82.9%, la empatía que hace parte de las competencias comunicativa y actitudinal presentó el 97.6% de acierto.

Las menores puntuaciones que hicieron parte de la competencia praxiológica en este grupo fueron velocidad de infusión con 36.6%, volumen de infusión 41.5% y efecto adverso 43.9 % de respuestas acertadas. Para estas dos primeras variables, el estudiante requiere efectuar cálculos y resolver problemas matemáticos. Este requisito junto con expresión oral, escrita, habilidades básicas de lectura y comprensión, son las limitantes más grandes para el estudiante de enfermería y se consideran como discapacidades del aprendizaje que muchas veces pasan desapercibidas por el profesor(22).

Si bien no se encontró diferencia en los puntajes pre y post de los sujetos de intervención y control, es cierto que la diferencia de puntajes fue estadísticamente mayor en el grupo de intervención, es decir se acercaron más a la calificación máxima que correspondía a 50.

Después de utilizar la estrategia de enseñanza en los dos grupos, se encontró una media de 31.4, con mínimo de 8 y máximo 42 para el grupo control y media de 36.7 con mínimo de 20 y máximo 50 en el grupo de intervención. Lo cual indica que la estrategia mejoró significativamente el desarrollo de competencias en comparación con la enseñanza tradicional, se concluye que la experiencia clínica contribuyó a mejorar el rendimiento.

**Figura 2:**
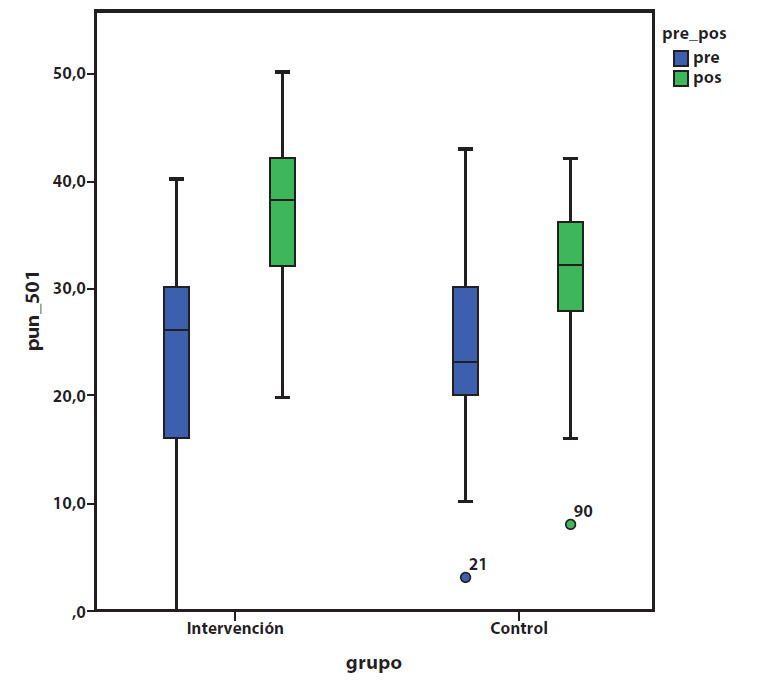
Representación Gráfica de la Calificación en Cada Grupo.

En la Figura 2 se observan los puntajes obtenidos en la prueba en los dos grupos. La mediana de la puntuación de los alumnos antes de impartir la clase en el grupo control fue de 23.8 con un mínimo de 2 y máximo de 43. Mientras que en el grupo intervención su mediana fue 22.5 con un mínimo de 0 y un máximo de 40.

De acuerdo con los resultados satisfactorios obtenidos con el OVA, se continuó aplicando la herramienta y durante el año 2020 con ocasión de la pandemia, resultó de gran utilidad y apreciación positiva por parte de los estudiantes de enfermería.

Fuente: Elaboración propia

## Discusión

Al comparar las características de las mediciones en el grupo control e intervención, se encontraron similares hallazgos por Lee y Lin(11), en donde los dos grupos demostraron una proporción significativamente mayor de sujetos que realizan la actividad de forma correcta. Sin embargo, la confianza aumentó para el grupo de intervención y disminuyó para el grupo de control. Resultados inferiores a 0,05 muestran que hay diferencias en los grupos, resultados mayores indican que no. Las dimensiones con mayor acierto en el grupo control fueron, hora correcta que pertenece a la competencia praxiológica con 92.5%, empatía 90% y liderazgo y trabajo en equipo 87.5% que corresponde a las competencia comunicativa y actitudinal. Con estas habilidades se aprende a resolver problemas interactuando y adquiriendo conocimientos entre compañeros y estas experiencias preparan a los estudiantes para la práctica de enfermería en la vida real(22).

La competencia con menor puntuación en el grupo control fue efecto adverso (25%), comparado con el grupo intervención 43.9%. Todavía es insuficiente la educación permanente sobre efectos adversos en medicación(10). De ahí la importancia de sensibilizar a los educadores en enfermería para que enfaticen la enseñanza en la identificación de esta condición en niños e incentivar a los profesionales sobre la importancia de la notificación en la sospecha de este problema, la educación continua es un instrumento esencial para propender con una atención segura en el paciente.

En el análisis general por competencias, se encontró que los mejores aciertos se presentaron en la evaluación de la competencia comunicativa y actitudinal, seguida por la cognitiva y por último la praxiológica. Al respecto, se contrasta con un estudio en el que las puntuaciones más altas se dieron en las habilidades prácticas en comparación con las que medían conocimientos(23).

La estrategia mejoró significativamente el desarrollo de competencias en comparación con la enseñanza tradicional. Este hallazgo es parecido a un estudio realizado en el cual al efectuar el análisis estadístico en el grupo intervención, el ECOE tuvo resultados superiores(24) con lo cual se concluye que la experiencia clínica contribuyó a mejorar el rendimiento. Se destaca la importancia de desarrollar estrategias de enseñanza que fomenten la comprensión conceptual(18).

La competencia comunicativa en la variable empatía con el cuidador, presentó altos porcentajes de respuesta correcta en el grupo intervención (97.6%). Este concepto se enfoca en deshacerse de los hábitos de comunicación que se centran en sí mismo para ponerse en el lugar de la otra persona, en este caso el cuidador. Se espera que los estudiantes integren la empatía con el esfuerzo para escuchar al otro. No se trata de influenciarlo sino basado en sus experiencias, se busca la comprensión y solidaridad con el ser humano(25). Si bien se obtuvieron resultados bastante favorables en esta variable para los dos grupos, es importante analizar que en la enseñanza tradicional en ocasiones no se transfieren estas habilidades, al encontrar que menos del 27% de los recién graduados en enfermería se sienten seguros de comunicarse con los pacientes y sus familias, así como con equipo de salud(26).

Llama la atención la competencia liderazgo y trabajo en equipo, en la cual el grupo control estuvo mejor valorado con el 87.5%, mientras que el grupo intervenido obtuvo 70.7%. De todas maneras, se resalta que estas variables promueven el rendimiento del futuro profesional, puesto que cuando la enfermera trabaja con colegas dentro de la misma organización, se enaltece la excelencia en el desempeño. Asimismo, surge un impacto positivo en la participación de los estudiantes y en los modelos a seguir(27) y el trabajo en equipo mejora la seguridad del paciente(28)-(29).

En época de pandemia ocasionada por Covid-19, la aplicación del objeto virtual favoreció el proceso enseñanza aprendizaje en estudiantes del curso de Cuidado de la Salud al Niño quienes manifestaron que la estrategia resultó ser interesante, participaron de manera estratégica y organizada cumpliendo en cada etapa con las tareas propuestas, manejando su tiempo e interactuando de manera efectiva con los compañeros. Lo anterior coincide con Parra(4) quien destaca la amplia motivación académica que se obtuvo frente a la capacidad que tenían los estudiantes para evaluar los módulos de aprendizaje. Cabe destacar que como en el estudio de Silva(4), el OVA permitió evaluar el conocimiento de manera relajada, sin comprometerse a la presión de una evaluación formal de la competencia asociada, pero a la vez fomenta la responsabilidad individual y resulta ser especialmente útil al brindar la oportunidad de resolver problemas del área clínica(30),(31).

El periodo de emergencia sanitaria decretado a nivel mundial ha conllevado al uso de este tipo de estrategias para desarrollar las clases, el docente universitario ha tenido la oportunidad de fortalecer sus competencias digitales al entorno que la crisis internacional le ha propuesto como nuevo escenario educativo(3). De esta manera las metodologías se adaptaron al contexto y lograron sostener el aprendizaje en el entorno clínico de manera provisional como una oportunidad para enriquecer el proceso enseñanza-aprendizaje y desarrollar actividades innovadoras(1),(2).

### Limitaciones

La enseñanza mediada por objetos virtuales de aprendizaje, todavía constituye un desafío para el docente investigador, es pertinente el desarrollo de otros estudios en diferentes contextos de atención. Así como el análisis de los aspectos relacionados con el proceso de enseñanza-aprendizaje con la utilización del OVA y su impacto en la formación de futuros profesionales de enfermería, con el fin de contribuir al conocimiento científico en esta área.

En cuanto al aspecto organizacional, al inicio el ECOE presentó algunos obstáculos en el diseño por el número elevado de estudiantes, la cantidad de docentes necesaria y la logística requerida para la aplicación de la prueba, lo cual implica alto costo económico, tiempo y recursos humanos. Lo ideal sería utilizar pacientes reales. Sin embargo, por cuestiones éticas y costos del talento humano, no se utilizaron niños con sus padres verdaderos, únicamente simuladores y actores que ejercieron el papel de cuidador.

En algunas ocasiones, la movilización de emociones y el estrés generado en el estudiante durante el ECOE, influyó en el desempeño. Algunos profesores no lograron identificar entre el bajo rendimiento causado por factores relacionados con el estrés y el generado por niveles bajos de competencia clínica.

Aunque los alumnos programados a la mitad o final de la jornada podían disponer de información previa al inicio de su prueba, no se puede afirmar que haya existido un efecto de aprendizaje mayor por la experiencia de los compañeros que anteriormente presentaron en examen.

## Conclusiones

Se determinó la efectividad del objeto virtual de aprendizaje al favorecer el proceso enseñanza aprendizaje en estudiantes del curso de Cuidado de la Salud al Niño de quinto semestre del programa de enfermería de la Universidad de los Llano. Se logró probar que la enseñanza y la práctica basada en evidencia son instrumentos de gran utilidad para el cuidado de enfermería teniendo en cuenta que la formación profesional es esencial para brindar cuidado seguro y de calidad. En este cometido, el profesor tiene gran responsabilidad. Por tanto, la educación en enfermería debe estar enmarcada en la calidad y seguridad, buscando la excelencia en la práctica.

El uso del OVA permitió desarrollar la competencia de administrar medicamentos en pediatría, de una forma crítica y reflexiva, ahora puede ser utilizado las veces que sea necesario, es un método flexible que va al ritmo que el estudiante decida, se ajusta a su estilo de aprendizaje y a la disponibilidad de su tiempo. Esto permite mayor autonomía, responsabilidad y disciplina.
